# Current tuberculin reactivity of schoolchildren in the Central African Republic

**DOI:** 10.1186/s12889-015-1829-8

**Published:** 2015-05-17

**Authors:** Fanny Minime-Lingoupou, Rock Ouambita-Mabo, Aristide-Désiré Komangoya-Nzozo, Dominique Senekian, Lucien Bate, François Yango, Bachir Nambea, Alexandre Manirakiza

**Affiliations:** Institut Pasteur de Bangui, PO Box 923, Bangui, Central African Republic; Ministry of Public Health, Population and AIDS Control, PO Box 883, Bangui, Central African Republic

**Keywords:** Tuberculosis, Tuberculin reactivity, Central African Republic

## Abstract

**Background:**

The tuberculin skin test (TST) is the recommended method for screening for *Mycobacterium tuberculosis* infection in many countries. We used this technique to assess bacillus Calmette-Guérin (BCG) status and to estimate the current prevalence and annual rate of latent tuberculosis infection in schoolchildren in the Central African Republic.

**Methods:**

Two tuberculin units of 0.1 ml purified protein derivative TR23 were injected intradermally into the left forearm of 2710 children attending school in Bangui and Ombella M’Poko. The induration size was interpreted at cut-off points of ≥5 mm, ≥10 mm and ≥15 mm. The annual infection rate was estimated as the average number of infections in the study sample each year between birth and the time of the survey.

**Results:**

Overall, there was no reaction to the TST (no induration) in 71.7 % (95 CI, 68.3–75.3 %) of BCG-vaccinated children and 82.9 % (95 CI, 74.1–91.4 %) of non-vaccinated children. The proportions of children who gave a TST reaction above ≥10 mm and ≥15 mm cut-off was 18.4 % (95 % CI, 16.8–20.1 %) and 8.9 % (95 % CI, 7.8–10.0 %), respectively. The proportions of TST reaction above these cut-offs were 19.6 % (95 % CI, 17.4–21.9 %) and 8.1 % (95 % CI, 6.7–9.6 %), respectively. The annual infection rate was 0.8 % at the cut-off point of ≥15 mm.

**Conclusion:**

This study provides updated data on rates of tuberculosis infection in the Central African Republic. It is remarkable that most of the children had negative tuberculin reactivity. More studies are required to understand the factors that determine the low tuberculin reactivity in this population.

## Background

In 2010, WHO reported that one third of the world’s population had latent tuberculosis (TB) infection, with 9.4 million new overt cases annually [1]. Children are important targets for the prevention of tuberculosis (TB) infection because they are at greatest risk for activation of their infections [2]. The tuberculin skin test (TST) is widely used in screening for latent *M. tuberculosis* infection and for determining induced hypersensitivity pre- and post-vaccination in schoolchildren [3–5].

In 2010 in the Central African Republic, mortality from TB was estimated at 50 per 100,000 population and the incidence at 367 per 100, 000 [6]. A national TB control programme was established in 1995 with the objective of reducing the incidence and prevalence of TB by 50 % before 2015. The directly observed therapy short course (DOTS) strategy was introduced in 2005; coverage reached 76 % in 2009, and 4305 new TB cases were notified [7]. A paediatric survey conducted in 2011 in Bangui showed a rate of positive sputum smear pulmonary TB of 23.5 % among 425 clinically suspected cases (G. Bobossi-Serengbe, unpublished data).

All neonates are vaccinated according to the BCG immunization policy at birth or within a few days of birth, as suggested by WHO [8]. BCG vaccination coverage in children was estimated at 72.4 % in 2010 [9]. The extent of TB infection in the Central African Republic has never been surveyed with the TST since establishment of the national programme, and the only data available on tuberculin reactivity in the country were reported in a survey conducted in 1988 in Bangui [10], which showed a prevalence of TB infection of 7.9 % and an estimated annual risk of 1.1 %. These figures rank the country among those with a low prevalence. These data are, however, old and refer only to Bangui. The study reported here provides recent data on TST outcomes in both Bangui, the capital, and also in the neighbouring rural area of Ombella M’Poko.

## Methods

### Survey area and population

**Fig. 1:**
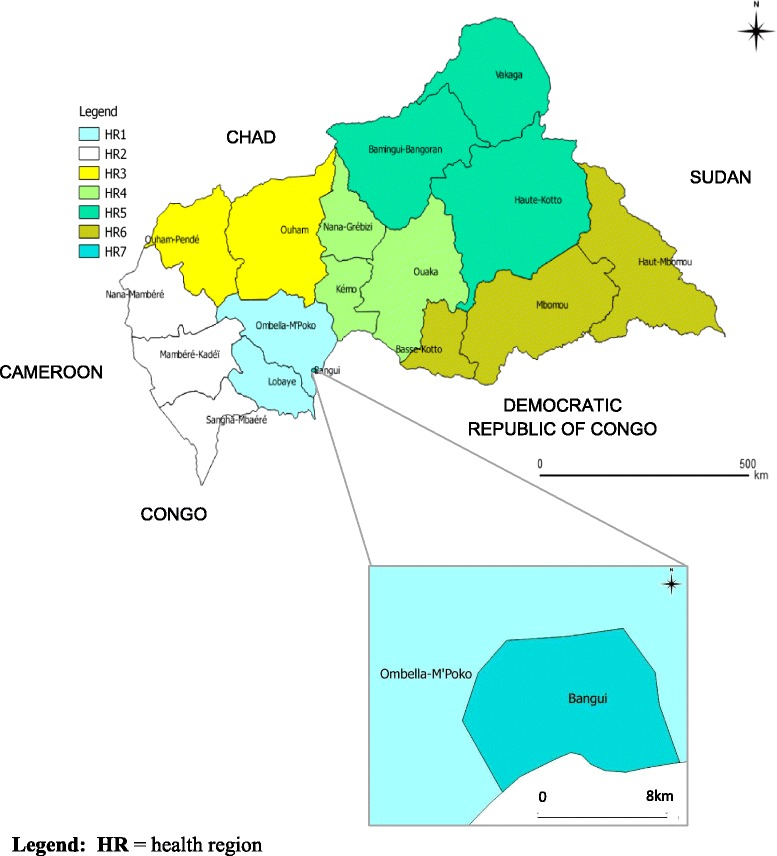
Location of study area: health regions 1 and 7. This map was created by the authors using the QGIS 2.2.0 software, from the Central African Republic shape file available at http://wwwn.cdc.gov/epiinfo/html/shapefiles.htm

As part of a policy to decentralize the health system, the Central African Republic was divided into seven health regions (Fig. 1). This survey was conducted in health regions one (Ombella M’Poko, a rural district) and seven (Bangui city, the capital of the Central African Republic) in 2011, with populations of 356,725 and 622,771 inhabitants, respectively (general population census, 2005). The TB rate is 134 per 100,000 population in Ombella M’Poko and 284 per 100,000 population in Bangui. The study population comprised primary schoolchildren aged 6–12 years.

### Ethics statement

The study protocol was approved by the ethics committee of the Central African Republic, and written consent for tuberculin testing and vaccination with BCG was obtained from the parents or guardians of eligible children.

### Sampling

A sample size of 1600 children was estimated for each region based on an expected TST reactivity rate of 75, for 3 % precision, a design effect of two, a minimum test read of 70 and at a 5 % significance level. A list of primary schools (clusters) and the population size of each school were obtained from the Ministry of Education. 57 schools (30 in Bangui and 27 in Ombella M’Poko) were included in the survey after probability proportional sampling based on the number of children attending each school. The survey was conducted between February and April 2011.

### Training

A team of six nurses received 4 weeks’ training in using and reading the TST according to the international protocol [11] during a pilot survey conducted in four schools in Bangui in January 2011.

### Tuberculin skin testing and data collection

Written consent for children’s participation in the study was obtained from the parents or legal guardians before the start of the study. A self-administered questionnaire was completed by all parents or guardians of children for age, sex, address, prior BCG vaccination as reported by parents or guardians or recorded on immunization cards, household contact with TB and history of chronic disease and fever. A clinical investigation was performed to detect any sign of active TB and scars of prior BCG vaccination. The exclusion criteria were: a history of chronic disease, symptoms of infectious disease (fever, cough, runny nose) that might interfere with TST reactivity, allergic disease and incomplete questionnaire at the time of screening.

The TST was performed, regardless of BCG scar status, with two tuberculin units of purified protein derivative TR23 in Tween, supplied by the Statens Serum Institute (Copenhagen, Denmark). A dose of 0.1 ml was injected intradermally into the left forearm. Skin reactions were read 72 h later, and the transverse diameter of induration was measured in millimeters with a transparent, flexible 15 mm ruler. Each induration was assessed by one reader; when there was doubt, a reference reader or the principal investigator did a second reading.

The guidelines of the national TB control programme state that BCG-vaccinated children with a TST result ≥15 mm and unvaccinated children with a result ≥10 mm who are household contacts of a TB case and have symptoms of TB should be treated with anti-TB drugs. Children with a TST result ≥15 or 10 mm without a household contact but with symptoms of TB are also investigated. For this survey, the size of the induration after TST was interpreted as follows: <5 mm was considered negative, 5–9 mm was interpreted as doubtful, and ≥10 mm was considered positive for a recent TB infection [12–14], and these children were referred to the clinic for investigation for TB.

### Statistical analysis

Data were double-entered into EpiInfo software version 3.5.3 (Centers for Disease Control and Prevention, USA). Statistical analysis was conducted with STATA 11.2.

The frequency distribution of induration sizes was plotted. Age was categorized as 6–9 and 10–12 years, and TST reaction induration size was categorized at cut-off points of ≥5 mm, ≥10 mm and ≥15 mm. The results for each categorical variable (gender, age category, health region and cut-off points) were presented as frequencies and proportions. We assessed whether the results differed according to BCG vaccination and without BCG scar status, and TST results (cut-off points of ≥5, ≥10 and ≥15 mm) were calculated as odds ratios (ORs) and their 95 % confidence intervals (CIs). The reaction induration size for each cut-off was considered to be latent TB infection if the effect of BCG vaccination on tuberculin reactivity was not statistically significant (OR significantly different from 1)*.* Proportions were compared in the chi-squared test. A *p* v alue <0.05 was considered statistically significant.

The annual risk for infection was calculated from the estimated prevalence of infection (P) calculated from the equation 1–(1–P)^1/A^, where A is the mean age of the tested children [15]. Indurations of ≥ 10 mm and ≥15 mm were selected as cut-off points to estimate the prevalence of infection and to calculate the annual risk for infection.

## Results

Of the 3280 children recruited for the survey, we excluded 174 who were absent at the time of testing, 87 who were absent at the skin test reading and 309 of unknown BCG status, leaving 2710 (82.6 %) children for the final analysis, comprising 1469 (54.2 %) in Bangui and 1241 (45.8 %) in Ombella M’Poko. No suspected cases of active TB were identified by clinical examination. Those who completed the TST reading and those who were absent at time of skin reading did not differ by gender or age. The proportions of children absent at TST was 8.5 % (116/1357) in Ombella M’Poko and 3.8 % (58/1527) in Bangui (*p* < 0.0001), and the proportions of children absent at skin reading was 4.4 % in Ombella M’Poko (57/1298) and 2.0 % in Bangui (30/1499) (*p* = 0.0004).

Table 1 shows the distribution of the study population according to BCG vaccination and BCG scar status by gender, age and health region. The proportions of non-vaccinated and vaccinated children did not differ by gender or age group (*p* = 0.873 and 0.139, respectively), but more vaccinated children were found in Bangui than in Ombella M’Poko (*p* = 0.014). More children aged 6–9 years than those aged 10–12 years had a BCG scar (*p* = 0.006).

**Table 1:** Distribution of gender, age and geographical area according to BCG vaccination and BCG scar status

Characteristics	Vaccinated			Vaccinated		
	Yes	No	*P*	Without BCG scar	BCG scar	*P*
				*n* (%)	*n* (%)	
	*n* (%)	*n* (%)				
Gender						
Male	1111 (83.7)	216 (16.3)	0.873	371 (33.4)	740 (66.6)	0.508
Female	1161 (83.9)	222 (16.1)		403 (34.1)	758 (65.9)	
Age (years)						
6–9	979 (85.1)	172 (14.9)	0.139	303 (30.9)	676 (69.1)	0.006
10–12	1293 (82.9)	266 (17.1)		471 (36.4)	822 (63.6)	
Health region						
Bangui	1255 (85.4)	214 (14.6)	0.014	439 (35.0)	816 (65.0)	0.308
Ombella M’Poko	1017 (81.9)	224 (18.1)		335 (32.9)	682 (67.1)	

**Fig. 2:**
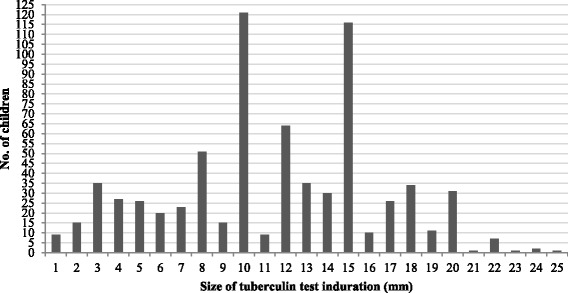
Distribution of TST induration (≥1 mm)

The BCG coverage rate was 83.8 % (2272/2710), and a BCG scar was observed in 65.9 % of children (95 % CI, 66.5–75.1 %). The plot of tuberculin reaction induration size showed a bimodal trend at 10 mm and 15 mm (Fig. 2). There was no reaction to the TST (no TST induration) in 71.7 % (95 % CI, 68.3–75.3 %) of BCG-vaccinated children and 82.9 % (95 % CI, 74.1–91.4 %) of non-vaccinated children.

The distribution of positive TST results at each cut-off value according to BCG scar status is shown in Table 2. The proportions of children with a TST reaction above each cut-off value were 23.4 % (95 % CI, 21.6–25.3 %) at ≥5 mm, 18.4 % (95 CI, 16.8–20.1 %) at ≥10 mm and 8.9 % (95 % CI, 7.8–10.0 %) at ≥ 15 mm. BCG vaccination status influenced the induration at ≥ 5 mm (OR, 1.8; 95 % CI, 1.4–2.4) and ≥ 10 mm (OR, 1.7; 95 % CI, 1.2–2.3) but not at ≥ 15 mm. There was no evidence that the proportions above the cut-offs were influenced by BCG scar status.

**Table 2:** Positive tuberculin skin test by induration size cut-off according to BCG scar status

Cut-off point (mm)	All		Without BCG scar		BCG scar		BCG effect	
	*n* (%)	95 % CI	*n* (%)	95 % CI	*n* (%)	95 % CI	OR^a^	95 % CI
5	634 (23.4)	[21.6–25.3]	182 (23.5)	[20.2–27.2]	384 (25.6)	[23.1–28.3]	1.1	[0.9–1.4]
10	499 (18.4)	[16.8–20.1]	151 (19.5)	[16.5–22.9]	293 (19.6)	[17.4–21.9]	1.0	[0.8–1–2]
15	240 (8.9)	[7.8–10.0]	84 (10.8)	[8.7–13.4]	121 (8.1)	[6.7–9.6]	0.7	[0.5–1.0]

^a^Crude odds ratio

The annual infection rate was 2.1 (95 % CI, 1.8–2.4) at the cut-off point of ≥ 10 mm and 0.9 (95 % CI, 0.8–1.1) at ≥ 15 mm in Bangui, and 1.8 (95 % CI, 1.5–2.1) and 0.7 (95 % CI, 0.5–0.8) in Ombella M’Poko, respectively. The overall rate was estimated to be 1.9 (95 % CI, 1.7–2.2) for ≥ 10 mm and 0.8 (95 % CI, 0.7–0.9) for ≥ 15 mm.

## Discussion

The proportions of children who had a TST reaction above the ≥ 10 mm and ≥ 15 mm cut-offs were 18.4 and 8.9 %, respectively, and the annual rate was 0.8 % above the ≥15 mm cut-off. A previous study conducted in Bangui in 1988 reported a prevalence of the TB infection of 7.9 ± 1.7 % and annual infection rate of 1.09 %. However, this previous study fixed the limit of positivity at 14 mm based to the bimodal distribution of the diameters TST induration diameter. Nevertheless, this profile of findings reflects that latent TB infection did not vary significantly during the period between these two studies.

BCG vaccination status had no effect on tuberculin reactivity (OR not significantly different from 1), which justifies use of the method for estimating prevalence and infection rate regardless of vaccination status. Many studies in other countries have demonstrated the usefulness of the TST for screening for TB infection regardless of BCG status, and there is growing consensus that the effect of neonatal vaccination with BCG wanes after about 5 years [16–18]. There is persistent reluctance, however, to agree that BCG vaccination induces sensitivity to tuberculin that might interfere with interpretation of TST results [19–21]. In our survey, the effect of having a BCG scar on TST reactivity was not statistically significant at the cut-off of ≥10 mm, and the proportion of BCG-vaccinated children who were TST negative was high. This finding is consistent with those of other studies [13, 16, 22].

The finding that the distribution profile of TST induration had a bimodal distribution at 10 and 15 mm indicates circulation of non-typical mycobacteria (NTM). A distribution with a higher proportion of lower reaction sizes is expected when cross-reaction with NTM is present [13]. Unfortunately, data on the prevalence of NTM in the Central African Republic is not available. In countries where the prevalence of NTM is high, a threshold value of 10 mm overestimates the prevalence of *M. tuberculosis* infection because of cross-sensitivity [23]. Hence, the induration threshold of ≥15 mm may be suitable for estimating the prevalence of infection and for calculating the annual risk for infection in the country.

This study has various limitations. The main one is that schoolchildren may not be representative of all children of school age in the country. Although accurate information on school attendance in the Central African Republic is not available, it is assumed to be low in rural areas (i.e. Ombella M’Poko). Children who are not at school are at higher risk for TB, because they often live in families of lower socio-economic status. Another limitation is that TST has low sensitivity in immunocompromised patients and in those with nutritional deficiency. The children in our survey were not tested for HIV infection. A study conducted between April 1998 and June 2000 reported that TB and HIV co-infection among children hospitalized in Bangui was 25.7 % (95 % CI, 20.7–31.2 %) [24]. The burden of TB in children is expected to be higher in high-incidence countries affected by the HIV epidemic [25]. The participants of this study were born between 1999 and 2005, while a study conducted in antenatal clinics in 2002 found a prevalence of HIV infection of 15 % among pregnant women [26]. As the transmission mother-to-children program was implemented in 2004, we expected that the majority of children were at high risk of HIV transmission (approximately 30 %). However, it is considered that the mortality observed among infected infants is very high [27, 28], and it is likely that very few these children might be HIV-infected in this study.

We did not study nutritional status; however, as there are poor socioeconomic conditions in the country, nutritional deficiency may have resulted in an underestimate of the TST reaction in this study. Better methods for detecting *M. tuberculosis*-specific antigen are emerging, including interferon gamma-release assays [29–31], even though negative results have been found with these assays with TST positive results [32], and there are significant differences in the performance of the two assays at ≥10 mm and ≥15 mm cut-offs [33]. Another limitation of this study is that information on BCG vaccination was based on recall by parents and guardians, who may not give accurate information on the BCG status of their children.

## Conclusion

This survey showed that a relatively high proportion of schoolchildren had negative tuberculin reactivity, irrespective of their BCG status. We provide updated data on the prevalence of TB infection and the annual risk for this infection. More studies are needed to understand the factors involved in the low tuberculin reactivity of this population, with more sustained TB control activity. It would be valuable to confirm these results in other parts of the Central African Republic before the conclusions can be generalized.

## References

[1] WHO Report (2010). Global Tuberculosis Control.

[2] Besser RE, Pakiz B, Schulte JM, Alvarado S, Zell ER, Kenyon TA (2001). Risk factors for positive mantoux tuberculin skin tests in children in San Diego, California: evidence for boosting and possible foodborne transmission. Pediatrics.

[3] Watkins RE, Brennan R, Plant AJ (2000). Tuberculin reactivity and the risk of tuberculosis: a review. Int J Tuberc Lung Dis.

[4] Pelly H (1991). The natural tuberculin conversion rate in the Western Health Board region: a review 1980–1986. Ir Med J.

[5] Lee SS, Chou KJ, Dou HY, Huang TS, Ni YY, Fang HC (2010). High prevalence of latent tuberculosis infection in dialysis patients using the interferon-gamma release assay and tuberculin skin test. Clin J Am Soc Nephrol.

[6] CAR (2010). Central African Republic, Ministry of Health. Annual Health Report.

[7] CAR (2006). Ministère de la Santé: Plan National de Dévéloppement Sanitaire.

[8] WHO (1995). Global tuberculosis programme and global programme on vaccines. Statement on BCG revaccination for the prevention of tuberculosis. Wkly Epidemiol Rec.

[9] Enquête par grappes à indicateurs multiples – MICS couplée avec a sérologie VIH, République centrafricaine, 2010. Assessed on 20january 2014 at http://www.childinfo.org/files/MICS4_CAR_FinalReport_2010_Fr.pdf.

[10] Sarda J, Monges J, Pujol C, Ndoyo J, Samba M, Monges P (1993). [Evaluation of the level of endemic tuberculosis in a survey of Banqui (Central African Republic)]. Med Trop (Mars).

[11] KNCV (2007). Generic protocol for tuberculin school survey. Royal Netherlands Tuberculosis Foundation.

[12] WHO (1963). Standard tuberculin test.

[13] Addo KK, van den Hof S, Mensah GI, Hesse A, Bonsu C, Koram KA (2010). A tuberculin skin test survey among Ghanaian school children. BMC Public Health.

[14] [Tuberculin intradermal reaction (IDR) or tuberculin test]. Med Mal Infect 2004, 34(8–9):358–63 15622978

[15] Cauthen GM, Pio A, tenDam HG (1988). Annual risk of tuberculous infection.

[16] Raharimanga V, Ratovoson R, Ratsitorahina M, Ramarokoto H, Rasolofo V, Talarmin A (2012). Tuberculin reactivity in first-year schoolchildren in Madagascar. Trop Med Int Health.

[17] Gulnar SB, Bulut BU (1997). Influence of BCG vaccination on tuberculin reactivity in healthy Turkish school children. Acta Paediatr.

[18] Mudido P, Guwatudde D, Nakakeeto M, Bukenya G, Nsamba D, Johnson J (1999). The effect of bacille Calmette-Gue´rin vaccination at birth on tuberculin skin test reactivity in Ugandan children. Int J Tuberc Lung Dis.

[19] Lohiya GS, Lilia TF, Krishna V (2009). Interpretation of the tuberculin skin test. J Natl Med Assoc.

[20] Sleiman R, Al-Tannir M, Dakdouki G, Ziade F, Assi NA, Rajab M (2007). Interpretation of the tuberculin skin test in bacille Calmette-Guerin vaccinated and nonvaccinated school children. Pediatr Infect Dis J.

[21] Kiwanuka JP (2005). Interpretation of tuberculin skin-test results in the diagnosis of tuberculosis in children. Afr Health Sci.

[22] Gopi PG, Subramani R, Nataraj T, Narayanan PR (2006). Impact of BCG vaccination on tuberculin surveys to estimate the annual risk of tuberculosis infection in south India. Indian J Med Res.

[23] Roelsgaard E, Iversen E, Blocher C (1964). Tuberculosis in tropi-cal Africa. Bull World Health Organ.

[24] Bobossi-Serengbe G, Tembeti PJ, Mobima T, Yango F, Kassa-Kelembho E (2005). [Tuberculosis and HIV co-infection among children hospitalized in Bangui (Central African Republic)]. Arch Pediatr.

[25] Middelkoop K, Bekker LG, Myer L, Dawson R, Wood R (2008). Rates of tuberculosis transmission to children and adolescents in a community with a high prevalence of HIV infection among adults. Clin Infect Dis.

[26] Matsika-Claquin MD, Massanga M, Menard D, Mazi-Nzapako J, Tenegbia JP, Mandeng MJ (2004). HIV epidemic in Central African Republic: high prevalence rates in both rural and urban areas. J Med Virol.

[27] Venkatesh KK, de Bruyn G, Marinda E, Otwombe K, van Niekerk R, Urban M (2011). Morbidity and mortality among infants born to HIV-infected women in South Africa: implications for child health in resource-limited settings. J Trop Pediatr.

[28] Newell ML, Coovadia H, Cortina-Borja M, Rollins N, Gaillard P, Dabis F (2004). Mortality of infected and uninfected infants born to HIV-infected mothers in Africa: a pooled analysis. Lancet.

[29] Thomas B, Pugalenthi A, Patel H, Woltmann G, Bankart J, Hoskyns W (2011). Concordance between tuberculin skin test and interferon-gamma assay and interferon-gamma response to mitogen in pediatric tuberculosis contacts. Pediatr Pulmonol.

[30] Mendez-Echevarria A, Gonzalez-Munoz M, Mellado MJ, Baquero-Artigao F, Vecino R, Perez E (2011). Optimizing interpretation of the tuberculin test using an interferon-gamma release assay as a reference standard. Pediatr Infect Dis J.

[31] Legesse M, Ameni G, Mamo G, Medhin G, Bjune G, Abebe F (2011). Community-based cross-sectional survey of latent tuberculosis infection in Afar pastoralists, Ethiopia, using QuantiFERON-TB Gold In-Tube and tuberculin skin test. BMC Infect Dis.

[32] Connell TG, Ritz N, Paxton GA, Buttery JP, Curtis N, Ranganathan SC (2008). A three-way comparison of tuberculin skin testing, QuantiFERON-TB gold and T-SPOT.TB in children. PLoS One.

[33] Tieu HV, Suntarattiwong P, Puthanakit T, Chotpitayasunondh T, Chokephaibulkit K, Sirivichayakul S (2014). Comparing interferon-gamma release assays to tuberculin skin test in Thai children with tuberculosis exposure. PLoS One.

